# The effect of chemotherapy on subjective cognitive function in younger early-stage breast cancer survivors treated with chemotherapy compared to older patients

**DOI:** 10.1007/s10549-019-05149-4

**Published:** 2019-02-12

**Authors:** M. L. Gregorowitsch, A. Ghedri, D. A. Young-Afat, R. Bijlsma, I. O. Baas, C. van Schaik-van de Mheen, M. J. Agterof, E. Göker, D. ten Bokkel Huinink, H. J. G. D. van den Bongard, H. M. Verkooijen, UMBRELLA study group

**Affiliations:** 1 Department of Radiation Oncology, University Medical Center (UMC) Utrecht, Heidelberglaan 100, 3584 CX Utrecht, The Netherlands; 2 University of Utrecht, Utrecht, The Netherlands; 3 Department of Epidemiology, Julius Center for Health Sciences and Primary Care, University Medical Center, Utrecht, The Netherlands; 4 Department of Medical Oncology, University Medical Center Utrecht, Utrecht, The Netherlands; 5 Department of Medical Oncology, Meander Medical Center, Amersfoort, The Netherlands; 6 Department of Medical Oncology, St. Antonius, Nieuwegein, The Netherlands; 7 Department of Medical Oncology, Alexander Monro Clinics, Bilthoven, The Netherlands; 8 Department of Medical Oncology, Diakonessenhuis, Utrecht, The Netherlands; 9 Imaging Division, University Medical Center Utrecht, Utrecht, The Netherlands

**Keywords:** Breast cancer, Chemotherapy, Cognitive function, Patient-reported outcome

## Abstract

**Purpose:**

To evaluate the impact of chemotherapy on subjective cognitive functioning according to age in a large cohort of breast cancer patients.

**Methods:**

Within the UMBRELLA cohort, 715 patients with early-stage primary invasive breast cancer (T1-3N0-1M0) were selected. Subjective cognitive function was assessed by means of the EORTC QLQ-C30 up to 24 months and compared between patients treated with and without chemotherapy, for three different age strata (355 patients < 55 years, 240 patients aged 55–65 years, and 120 patients > 65 years). Differences between chemotherapy and non-chemotherapy patients by age at different time points were assessed by linear mixed-effect models correcting for age, tumor stage, educational level, endocrine therapy, anxiety, and depression.

**Results:**

In total, 979 patients from the UMBRELLA cohort were included, of which 715 (73%) responded to baseline and at least one follow-up questionnaire. Questionnaire response rates ranged between 92 and 70%. The proportion of patients treated with chemotherapy decreased with age: 64% (*n* = 277) in patients < 55 years, 45% (*n* = 107) in patients 55–65 years, and 23% (*n* = 27) in patients > 65 years. Chemotherapy was associated with reduced subjective cognitive functioning. The impact of chemotherapy on subjective cognitive function was most pronounced in patients < 55 years, followed by those between 55 and 65 years. In the youngest age groups, patients treated with chemotherapy had significantly lower cognitive functioning up to 24 months. In women over 65 years, subjective cognitive functioning was comparable between patients treated with and without chemotherapy.

**Conclusion:**

This study confirms that chemotherapy is associated with impaired subjective self-reported cognitive functioning in breast cancer patients, and the effect persists at least up to 2 years after diagnosis. The impact of chemotherapy on self-reported cognitive functioning in the first 24 months is most pronounced in younger patients, especially those under 55 years of age.

**Electronic supplementary material:**

The online version of this article (10.1007/s10549-019-05149-4) contains supplementary material, which is available to authorized users.

## Introduction

In the past decades, advances in early tumor detection, improved surgery, and more effective adjuvant treatment have led to a decreased mortality from breast cancer [1]. As the number of breast cancer survivors is consistently increasing, long-term toxicity and morbidity following treatment are a growing concern among patients and physicians [2, 3].

Adjuvant chemotherapy reduces breast cancer-related mortality [6]. However, chemotherapy is also the prime suspect to cause cancer-related cognitive decline [4–6]. This condition encompasses a range of symptoms including memory loss, inability to concentrate, difficulty in thinking, and other subtle cognitive changes [8]. Incidence rates of cognitive symptoms vary, but it has been suggested that up to 71% of breast cancer patients suffer from some degree of cognitive impairment after chemotherapy [3, 7, 8]. For some patients, symptoms of reduced cognitive function may be transient, whereas for others it may persist and seriously impact quality of life [9, 10]. Deterioration in cognitive function is of particular importance in younger breast cancer patients, given their longer life expectancy and the detrimental effect of cognition on employment opportunities and daily activities (e.g., taking care of children) [9, 10].

Estimating chemotherapy-induced risks of cognitive dysfunction is important to guide clinical and shared decision making, to adequately inform patients, and to offer targeted rehabilitation programs to those most at risk of developing cognitive problems. Numerous studies have aimed to analyze impairment in cancer survivors using hard endpoints, but only few studies have compared patient-reported cognitive function in breast cancer patients and normative population [11].

The aim of the present study was to evaluate self-reported subjective cognitive function in early-stage breast cancer patients in different age groups up to 2 years after treatment, and to compare outcomes between patients treated with and without chemotherapy.

## Methods

This study conducted with data from the Dutch prospective observational breast cancer cohort UMBRELLA (Utrecht cohort for Multiple BREast cancer intervention studies and Long-term evaLuAtion) [12]. UMBRELLA was approved by the Medical Ethics Committee of the University Medical Center (UMC), Utrecht, the Netherlands, and is registered on clinicaltrials.gov (NCT02839863). The cohort includes patients (≥ 18 years) with histologically proven ductal carcinoma *in situ* or invasive breast cancer, who were referred to the Department of Radiation Oncology of the UMC, Utrecht, the Netherlands. All participants provided informed consent for the use of their clinical data and the collection of patient-reported outcomes (PRO) via standardized questionnaires. Informed consent was obtained after breast cancer surgery, before the start of radiotherapy, or in a minority of the patients during the course of neoadjuvant systemic treatment. For this study, we identified all adult patients ≤ 70 years of age, enrolled between October 2013 and September 2017 and who responded to at least two questionnaires assessing PROs including baseline questionnaire (i.e., responders, response rates: 70–92%). Patients were excluded when they had been diagnosed with a ductal carcinoma *in situ*, high-risk disease (cT4N2-3) or with clinical metastasis (M1).

All patients were treated according to the Dutch guidelines for breast cancer treatment [13]. When chemotherapy was indicated, patients were treated with (neo)adjuvant anthracycline-based chemotherapy and/or taxane-based chemotherapy. In case of Her2Neu receptor positivity, trastuzumab (± pertuzumab) was given. All participants had undergone mastectomy or breast-conserving surgery combined with axillary staging and/or surgery. All patients were scheduled for radiotherapy of the breast or chest wall, including regional radiotherapy if indicated. Adjuvant endocrine therapy was administered when indicated in hormone receptor positive patients.

### Data collection

Patient, tumor, and treatment characteristics were provided by the Netherlands Cancer Registry (NCR) of the Netherlands Comprehensive Cancer Organization (IKNL) [14]. Data on subjective cognitive functioning were assessed using the cancer quality-of-life core questionnaire of the European Organization for Research and Treatment of Cancer (EORTC QLQ-C30) [15]. The EORTC QLQ-C30 subscale for cognitive function includes two, 4-point Likert-scored items: (1) “Have you had difficulty in concentrating on things, like reading a newspaper or watching television?”; and (2) “Have you had difficulty remembering things?” [EORTC Cognitive Functioning (EORTC-CF)]. Anxiety and depression were assessed using the Hospital Anxiety and Depression Scale (HADS) [16]. Patient with scores of ≥ 8 were considered to have moderate of high probability of having anxiety or depressive disorders [17–19].

The EORTC QLQ-C30 questionnaire was administered at cohort entry (i.e., before radiotherapy, baseline) and at 3, 6, 12, 18, and 24 months thereafter. The HADS was administered at all aforementioned time points except at 3 months. Due to enrollment at the Radiation Oncology Department, a minority of the patients undergoing neoadjuvant chemotherapy already received treatment before cohort enrollment (i.e., baseline questionnaire). Questionnaires were collected within the Patient-Reported Outcomes Following Initial treatment and Long-term Evaluation of Survivorship registry (PROFILES) [20]. Subjective cognitive function scores of patients were compared with cross-sectional scores of an age-matched Dutch reference population including women without a history of breast cancer (total *n* = 944, Supplement Table 1) provided by PROFILES [20]. Data of the reference population were matched on age for the three different age strata (< 55 years *n* = 494, 55–65 years *n* = 247, > 65 years *n* = 203).

### Statistical analysis

Patients were categorized according to chemotherapy status (i.e., non-chemotherapy and chemotherapy group) to observe overall differences in subjective cognitive function. For this purpose, completion dates for questionnaires were compared with the dates of the start of (neo)adjuvant chemotherapy. Patients were categorized into the chemotherapy group when the start of chemotherapy preceded the date of completing the questionnaire. Therefore, patients were only categorized as chemotherapy patient, when chemotherapy had been started. For example, patients receiving adjuvant chemotherapy 4 months after baseline were coded as ‘non-chemotherapy group’ at baseline and 3 months, but as ‘chemotherapy group’ at 6, 12, 18, and 24 months. After 6 months, there were no more shifts from the non-chemotherapy to the chemotherapy group. All patients that had started chemotherapy, remained in the chemotherapy group. Next, patients were stratified by age in three groups, patients below 55 years, between 55 and 65 years, and above 65 years of age. Frequencies, proportions, and means with standard deviations for normally distributed variables (and medians with interquartile ranges (IQR) otherwise), were used to describe patient, tumor, and treatment characteristics.

The EORTC QLQ-C30 questionnaire was processed according to the EORTC scoring manual [21]. Cognitive function scores of the EORTC QLQ-C30 range from 0 to 100 with higher scores representing better cognitive functioning [21]. Changes in cognitive function within the non-chemotherapy and chemotherapy within different age strata were analyzed with linear mixed-effects models to account for the correlation within subjects between the repeated measurements [22].

Linear mixed-effects models tested the presence of an age group-by-time-by-treatment (chemotherapy treatment versus no chemotherapy treatment) interactions for subjective self-reported cognitive function. The model included a participant-specific random effect. Age, tumor stage, educational level, endocrine treatment, anxiety (HADS Anxiety score < 8 vs. ≥ 8), and depression (HADS Depression score < 8 vs. ≥ 8) were included as fixed effects to adjust for potential confounding effects. An autoregressive covariance structure of the first order (AR1) was used to define the correlations among observations, assuming correlations would be greater between measurements that were closer together in time compared with those further apart (i.e., exponential decline) [2]. Changes in subjective self-reported cognitive function are presented as the mean differences (MDs) with confidence intervals (95% CI), reflecting the difference between patients with chemotherapy and without chemotherapy treatment in the same age category group. The use of advanced modeling approaches allowed us to examine main effects and interactions between age, time, chemotherapy, and non-chemotherapy patients correcting for age, endocrine treatment, educational level, anxiety and depression, and tumor stage, handling the correlation structures of repeated measures nested within patients. In sensitivity analysis, we evaluated the effect of hormonal changes on subjective self-reported cognitive functioning. We included pre/perimenopausal and postmenopausal status at baseline instead of age to evaluate if the effect of chemotherapy would change, as age is related to subjective cognitive impairment. A second sensitivity analysis was performed excluding patients treated with neoadjuvant chemotherapy (i.e., only including adjuvant chemotherapy patients). We included only patients treated with adjuvant chemotherapy to exclude the effect of more advanced tumor stage and more intense treatment regime, which are most likely patients receiving neoadjuvant chemotherapy, on cognitive function.

As a measure of clinically meaningful difference, the standardized effect size (ES) was calculated (MD divided by the pooled standard deviation of the differences in scores) and classified as “no effect” (ES 0.2), “small effect” (ES, 0.2–0.4), “medium effect” (ES, 0.5–0.7), and “large effect” (ES ≥ 0.8), according to Cohen [23]. A decline of 10 points on the EORTC QLQ-C30 cognitive function scale was considered clinically meaningful [24, 25].

The level of statistical significance was *P* < 0.05 and corrections for multiple testing were made. Statistical analyses were performed using SPSS Statistics for Windows, version 23 (IBM Corp, Armonk, NY).

## Results

**Fig. 1:**
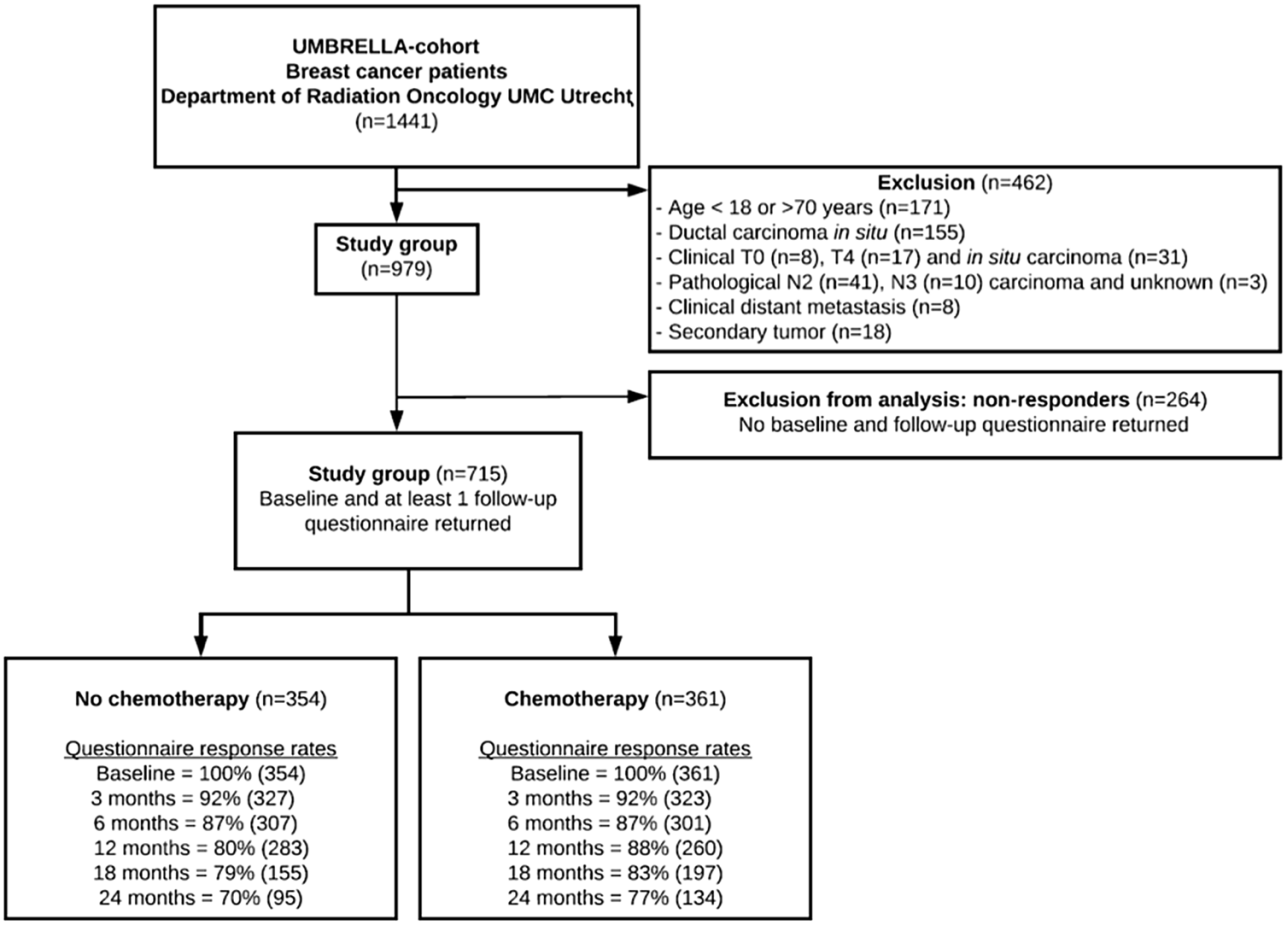
Flowchart of patient inclusion within the UMBRELLA breast cancer cohort and response rates. Response rates were calculated based on the opportunity patients had to return the questionnaire according to their inclusion date

In total, 1441 patients were enrolled in the cohort between October 2013 and September 2017 (Fig. 1). Of all participants, 979 patients met the inclusion criteria and 715 (73%) completed 2 or more quality-of-life (QOL) questionnaires. In total, 361 (50%) of the selected patients were treated with either neoadjuvant (*n* = 131, 35%) or adjuvant (*n* = 230, 65%) chemotherapy.

Patients in the chemotherapy group had a median age of 51 (IQR 13) and patients in the non-chemotherapy group had a median age of 60 (IQR 14, Table 1). Patients treated with chemotherapy more often presented with a higher pathological tumor stage (T2 or T3) and clinical positive lymph nodes and had undergone more extensive treatment (e.g., mastectomy, axillary lymph node dissection, and endocrine therapy).

**Table 1:** Demographics and disease characteristics of patients treated with and without chemotherapy in the first 24 months after enrollment in the UMBRELLA breast cancer cohort

	Chemotherapy	No chemotherapy
	No. of patients (%)	No. of patients (%)
Total no. of patients	361 (50)	354 (50)
Age in years at inclusion, median (IQR)	51 (13)	60 (14)
Age category		
< 55 years	227 (63)	128 (36)
55–65 years	107 (30)	133 (38)
> 65 years	27 (8)	93 (26)
Menopausal status at inclusion		
Premenopausal	158 (44)	58 (16)
Peri- or postmenopausal	183 (51)	269 (76)
Unknown	20 (6)	27 (8)
Pathological tumor stage		
T0	47 (13)	0 (0)
T1	181 (50)	316 (89)
T2	102 (28)	34 (10)
T3	20 (6)	0 (0)
Unknown	11 (3)	4 (1)
Course of chemotherapy treatment		
Neoadjuvant chemotherapy^a^	135 (37)	NA
Adjuvant chemotherapy	226 (63)	NA
Surgical treatment		
Breast-conserving surgery	278 (77)	344 (97)
Mastectomy	83 (23)	10 (3)
Most invasive axillary procedure		
Sentinel lymph node biopsy	284 (79)	343 (97)
Axillary lymph node dissection	60 (17)	5 (1)
Unknown	17 (5)	6 (2)
Estrogen receptor status		
Negative	98 (27)	12 (3)
Positive^b^	263 (73)	342 (97)
Unknown	0 (0)	2 (1)
HER2 receptor status		
Negative	270 (75)	338 (96)
Positive	91 (25)	6 (2)
Unknown	0 (0)	10 (3)
Type of chemotherapy		
Anthracycline based	328 (91)	NA
Non-anthracycline based	34 (9)	NA
Endocrine treatment		
No	109 (30)	218 (62)
Yes	252 (70)	135 (38)
Type of radiotherapy^c^		
Local radiotherapy	224 (62)	324 (92)
Locoregional radiotherapy^d^	137 (38)	29 (8)
Partial breast	0 (0)	1 (0)
Highest educational level^e^		
Secondary school ± elementary school	79 (22)	96 (27)
Lower vocational education	82 (23)	68 (19)
Community college	111 (31)	84 (24)
University	61 (17)	50 (14)
Unknown	28 (8)	56 (16)
Working tasks^e^		
Physical work	35 (10)	34 (10)
Mental work	145 (40)	105 (30)
Combination of physical and mental work	97 (27)	73 (20)
Unknown	119 (33)	142 (40)
HADS score on the Anxiety subscale, mean (SD)	6.2 (3.7)	5.5 (3.4)
HADS score on the Depression subscale, mean (SD)	3.7 (3.5)	3.1 (3.3)

All patients who received chemotherapy treatment in the first 24 months after enrollment in the UMBRELLA breast cancer cohort were considered chemotherapy patients and contributed to the chemotherapy group

Categories may not sum to total N or 100% because of missing values or rounding

*IQR* interquartile range, *NA* not applicable, *HER2* human epidermal growth factor receptor 2, *HADS* Hospital Anxiety and Depression scale

^a^Neoadjuvant chemotherapy was given in combination with immunotherapy if patients were HER2 receptor positive

^b^Estrogen receptor positive > 10%

^c^Radiotherapy on the breast or chest wall with or without boost on the tumor bed

^d^Includes radiotherapy on axillary and/or periclavicular lymph nodes and/or internal mammary nodes

^e^Self-reported

In total, half of the patients (*n* = 355) were aged 55 years or younger, 240 patients (33%) between 55 and 65 years and 120 patients (17%) were older than 65 years. The majority of patients (*n* = 227, 64%) in the youngest age group received chemotherapy, whereas 45% (*n* = 107) of the patients aged between 55 and 65 years and 23% (*n* = 27) of the > 65 years of age, were treated with chemotherapy (Table 2). Educational level was highest in patients aged < 55 years of age.

**Table 2:** Demographics and disease characteristics of patients below 55 years of age, between 55 and 65 years of age, and above the age of 65 years, participating in the UMBRELLA breast cancer cohort

	< 55 years	55–65 years	> 65 years
	No. of patients (%)	No. of patients (%)	No. of patients (%)
Total no. of patients	355 (50)	240 (34)	120 (17)
Total no. of patients treated with chemotherapy	227 (64)	107 (45)	27 (23)
Age at inclusion, median (IQR)	49 (6)	60 (4)	68 (2)
Menopausal status at inclusion			
Premenopausal	213 (60)	3 (1)	0 (0)
Peri- or postmenopausal	99 (28)	233 (97)	120 (100)
Unknown	43 (12)	4 (2)	0 (0)
Pathological tumor stage			
T0	32 (9)	12 (5)	3 (3)
T1	226 (64)	176 (73)	95 (79)
T2	71 (20)	45 (19)	20 (17)
T3	17 (5)	2 (1)	1 (1)
Unknown	9 (3)	5 (2)	1 (1)
Course of chemotherapy treatment			
Neoadjuvant chemotherapy^a^	102 (29)	25 (10)	7 (6)
Adjuvant chemotherapy	125 (35)	82 (34)	20 (17)
Surgical treatment			
Breast-conserving surgery	291 (82)	220 (92)	111 (93)
Mastectomy	64 (18)	20 (8)	9 (8)
Most invasive axillary procedure			
Sentinel lymph node biopsy	302 (85)	216 (90)	109 (91)
Axillary lymph node dissection	41 (12)	17 (7)	7 (6)
Unknown	12 (3)	7 (3)	4 (3)
Estrogen receptor status			
Negative	61 (17)	32 (13)	17 (14)
Positive^b^	294 (83)	207 (86)	102 (85)
Unknown	0 (0)	1 (0)	1 (1)
HER2 receptor status			
Negative	286 (81)	213 (89)	109 (91)
Positive	65 (18)	23 (10)	9 (8)
Unknown	4 (1)	2 (1)	1 (1)
Adjuvant chemotherapy treatment			
No	230 (65)	158 (66)	100 (83)
Yes	125 (35)	82 (34)	20 (17)
Type of chemotherapy			
Anthracycline based	207 (58)	99 (41)	23 (19)
Non-anthracycline based	21 (6)	8 (3)	5 (4)
Endocrine treatment			
No	144 (41)	115 (48)	69 (58)
Yes	211 (59)	125 (52)	51 (43)
Type of radiotherapy^c^			
Local radiotherapy	254 (72)	194 (81)	100 (83)
Locoregional radiotherapy^d^	101 (28)	45 (19)	20 (17)
Partial breast	0 (0)	1 (0)	0 (0)
Highest educational level^e^			
Secondary school ± elementary school	66 (19)	77 (32)	32 (27)
Lower vocational education	93 (26)	48 (20)	9 (8)
Community college	109 (31)	73 (30)	13 (11)
University	82 (23)	23 (10)	6 (5)
Unknown	5 (1)	19 (8)	60 (50)
HADS score on the Anxiety subscale, mean (SD)	6.2 (3.7)	5.7 (3.7)	5.2 (3.4)
HADS score on the Depression subscale, mean (SD)	3.6 (3.5)	3.3 (3.4)	3.1 (3.3)

Categories may not sum to total N because of missing values

*NA* not applicable, *HER2* human epidermal growth factor receptor 2, *HADS* Hospital Anxiety and Depression scale

^a^Neoadjuvant chemotherapy was given in combination with immunotherapy if patients were HER2 receptor positive

^b^Estrogen receptor positive > 10%

^c^Radiotherapy on the breast or chest wall with or without boost on the tumor bed

^d^Includes radiotherapy on axillary and/or periclavicular lymph nodes and/or internal mammary nodes

^e^Self-reported

### Comparison by treatment group

**Fig. 2:**
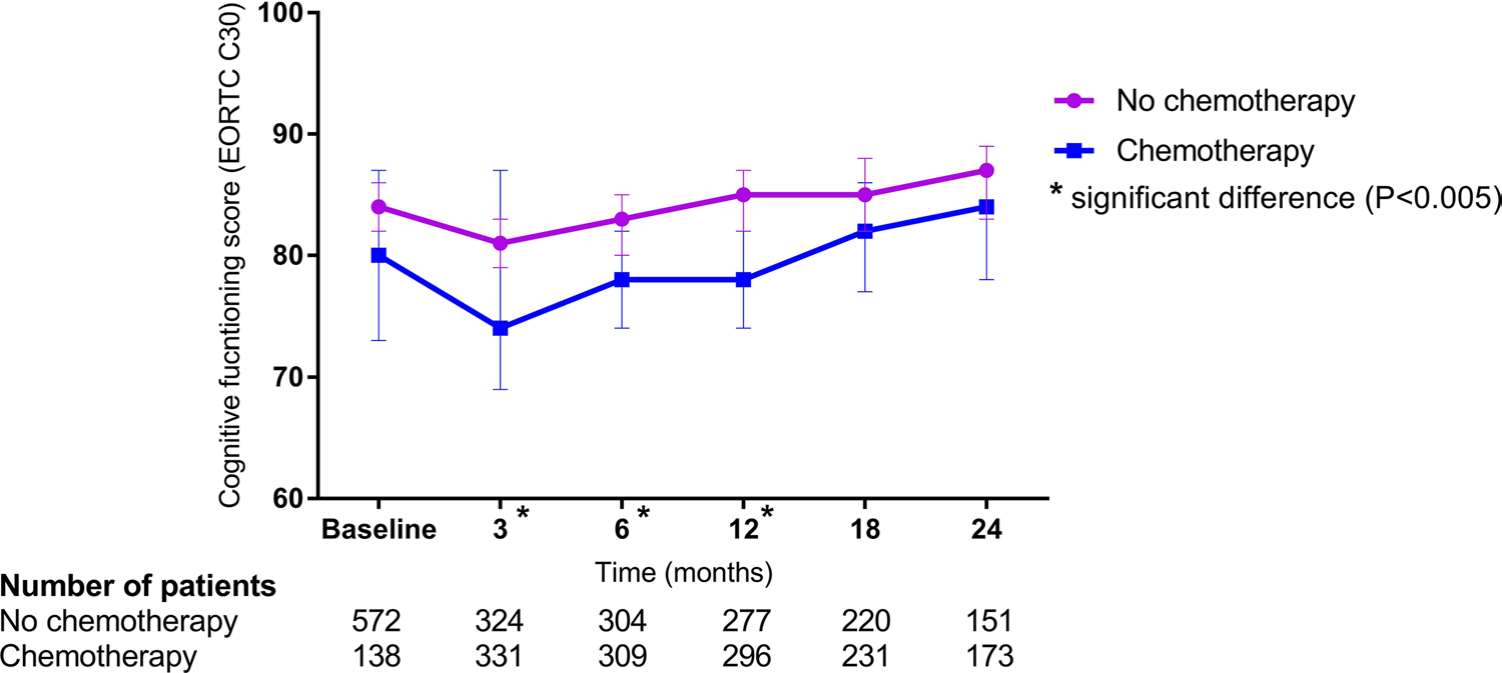
Cognitive functioning. Higher score indicates better cognitive functioning

Subjective self-reported cognitive functioning, adjusted for educational level, age, tumor stage, endocrine therapy, anxiety, and depression (HADS) of patients treated with chemotherapy, was significantly worse 3, 6, and 12 months after the start of radiotherapy compared to non-chemotherapy patients. In both groups, subjective self-reported cognitive function deteriorated at 3 months when compared to baseline scores and improved thereafter with scores comparable to baseline scores at 24 months (Fig. 2).

### Comparison by age and treatment group

**Fig. 3:**
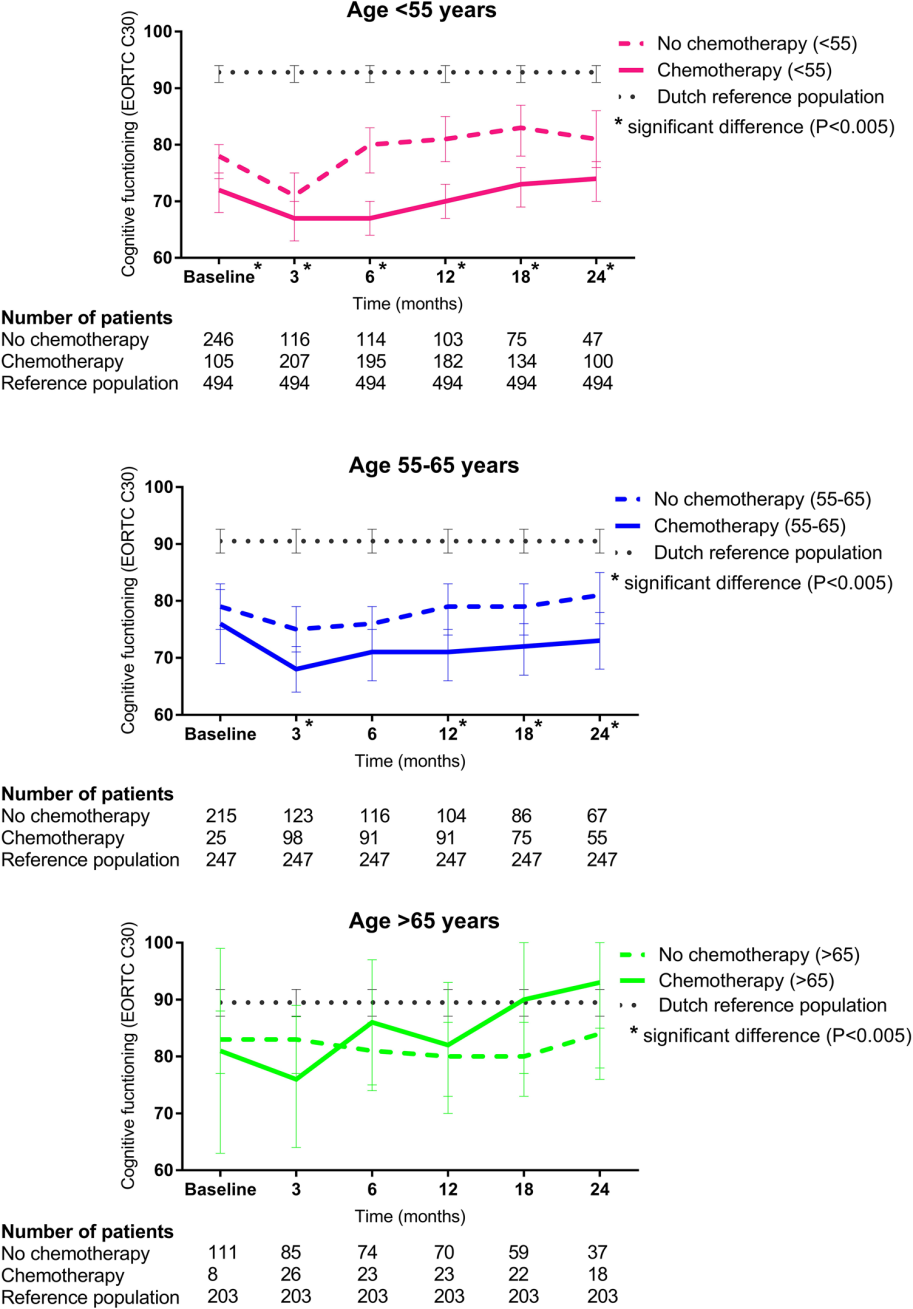
Cognitive function in early-stage breast cancer patients according to chemotherapy treatment stratified by age categories and compared to the age-matched Dutch reference population. Cognitive function was assessed with the cancer questionnaire of the European Organization for Research and Treatment of Cancer (EORTC QLQ-C30). Results are presented as adjusted mean scores accompanied with 95% confidence intevals. Higher score indicates better cognitive function

Younger patients (< 55 years) treated with chemotherapy reported significantly worse subjective self-reported cognitive functioning than patients from the same age who did not receive chemotherapy treatment (Fig. 3), after adjusting for age, tumor stage, educational level, endocrine therapy, anxiety, and depression (HADS) in mixed model analysis (Table 3). Mean differences (MD) in subjective self-reported cognitive functioning scores in patients < 55 years of age, treated with or without chemotherapy were largest at 6, 12, and 18 months (MD 12.3, 95% CI 8.2–16.3, MD 10.7, 95% CI 6.5–14.9, and MD 10.7, 95% CI 5.9–15.4, resp.). Effect sizes varied from 0.2 to 0.6, indicating a small/medium effect (Table 2). Compared to the age-matched Dutch reference population (*n* = 494), cognitive function of both the chemotherapy and non-chemotherapy patients was lower at all time points.

**Table 3:** Cognitive function in younger (< 55 years), middle-aged (55–65 years), and older (> 65 years) patients with breast cancer assessed with the EORTC QLQ-C30 at baseline, 3, 6, 12, 18, and 24 months after first consultation with the radiation oncologist

	Age < 55 years				Age 55–65 years				Age > 65 years				Overall *P* value^c^
No chemotherapy (*reference group*)	*n* = 128				*n* = 133				*n* = 93				
Chemotherapy	*n* = 277				*n* = 107				*n* = 27				
	MD^a^	95% CI	*P* value^b^	ES^c^	MD^a^	95% CI	*P* value^b^	ES^c^	MD^a^	95% CI	*P* value^b^	ES^c^	
Baseline	5.6	1.9–9.3	0.003	0.2	2.9	− 4.1 to 9.9	0.422	0.1	1.7	− 16.6 to 20.1	0.853	0.0	0.017
3 Months	4.4	0.5–8.2	0.024	0.2	6.8	2.2–11.2	0.003	0.3	7.7	− 4.3 to 19.7	0.208	0.3	
6 Months	12.3	8.2–16.3	0.000	0.6	4.4	− 0.3 to 9.1	0.072	0.2	− 4.7	− 16.8 to 7.5	0.454	0.2	
12 Months	10.7	6.5–14.9	0.000	0.5	8.1	3.1–13.0	0.001	0.4	− 1.8	− 14.2 to 10.5	0.772	0.1	
18 Months	10.7	5.9–15.4	0.000	0.5	7.0	1.6–12.3	0.010	0.3	− 9.9	− 23.3 to 3.6	0.151	0.4	
24 Months	7.4	1.8–13.2	0.009	0.4	8.0	2.0–13.9	0.009	0.4	− 9.5	− 25.1 to 6.1	0.235	0.3	

The differences in mean score (MD) between in the younger, middle-aged, and older patient group are adjusted for endocrine treatment and show the difference in cognitive function between chemotherapy and non-chemotherapy treatment

Patient-reported outcomes on cognitive functioning according to EORTC QLQ-C30

Between-group effects were assessed using mixed models including the measurements obtained at baseline and at 3, 6, 12, 18, and 24 months, adjusted for multiple testing, age, tumor stage, endocrine treatment, educational level, anxiety (HADS), and depression (HADS). Patients not exposed to chemotherapy serve as a reference category to calculate mean differences

*N* number of patients, *MD* mean difference, *95% CI* confidence interval, *ES* effect size

^a^Difference in mean score with reference group

^b^The *P* value shown for the difference between no chemotherapy and chemotherapy group

^c^Standardized effect size calculated (mean difference divided by the pooled standard deviation) as a measure for minimal clinically important change. Small effect if ES 0.2–0.4, medium 0.5–0.7, large effect > 0.8

^d^The *P* value shown for the interaction Age-by-time by treatment (no chemotherapy versus chemotherapy)

In patients aged between 55 and 65 years, adjusted scores of subjective self-reported cognitive function were significantly lower in those treated with chemotherapy at 3, 12, 18, and 24 months. Mean scores for subjective self-reported cognitive function were significantly worse at all time intervals except for baseline scores and 6 months (MD 6.8, 95% CI 2.2–11.2, MD 8.1, 95% CI 3.1–13.0, MD 7.0, 95% CI 1.6–12.3, MD 8.0, 95% CI 2.0–13.9, at 3, 12, 18, and 24 months, resp., Table 3). The subjective self-reported cognitive score of the age-matched Dutch reference population was higher.

Among patients above 65 years of age, the difference in cognitive function scores between chemotherapy groups was less pronounced (Fig. 3). At 3 months, patients treated with chemotherapy had lower score compared to non-chemotherapy patients (MD 7.7, 95% CI − 4.3 to 19.7, ES; 0.3, Table 3). At 6, 12, 18, and 24 months, self-reported subjective cognitive function scores were lower, but non-significant, in patients not treated with chemotherapy. The age-matched (> 65 years) Dutch reference population showed comparable scores in chemotherapy patients at 18 and 24 months.

Sensitivity analysis of chemotherapy on subjective self-reported cognitive function in pre/peri versus postmenopausal patients showed a similar effect of chemotherapy as in the effect of chemotherapy in patients < 55 years of age and patients between 55 and 65 years of age. The effect of chemotherapy on subjective cognitive function was largest (ES: 0.4–0.6) in pre/perimenopausal patients (Supplement Table 3). Sensitivity analysis on the effect of cognitive functioning only including patients who received adjuvant chemotherapy patients shows bigger difference in cognitive function in the youngest patient group at baseline (MD 12.9, 95% CI; 3.7–22.0 Supplement Table 4).

## Discussion

This prospective cohort study showed that self-reported subjective cognitive function within the first 2 years after breast cancer was lower in early-stage breast cancer patients treated with chemotherapy compared to patients treated without chemotherapy with significantly lower scores at 3, 6, and 12 months. The short- and long-term effect of chemotherapy on self-reported subjective cognitive function was most pronounced in patients younger than 55 years of age. Although self-reported subjective cognitive functioning of younger patients slightly improved 3 months after treatment, the chemotherapy group consistently reported statistically significant worse scores. Up to 2 years after diagnosis, younger patients (≤ 65 years) experienced worse self-reported subjective cognitive functioning compared to the Dutch reference population, matched on age reflecting the same age of the two youngest age strata (respectively, < 55 and 55–65 years). This was in contrast with older patients (> 65 years), in whom self-reported subjective cognitive function was found to be more similar between patients who were exposed to chemotherapy, not exposed to chemotherapy, and healthy controls.

Many cross-sectional and longitudinal studies have found evidence for chemotherapy-induced self-reported subjective cognitive changes in patients with breast cancer; however, studies on the effect in different age categories are limited [11, 26–28]. In several studies on risk factors for cognitive decline, age has been found to be a well-established factor for subjective and objective cognitive deterioration. Even though most studies report that older patients may be more vulnerable to toxic effects of treatment, we did not see an (strong) effect of chemotherapy on self-reported subjective cognitive function in patients aged over 65 years; however, the number of older patients treated with chemotherapy was small in this study [2, 29–33]. Longitudinal study in 112 breast cancer patients from Ahles et al. [32] found that older patients who were exposed to chemotherapy performed worse on neuropsychological measures of processing speed compared to non-chemotherapy patients and healthy controls, i.e., older patients had lower objective cognitive functioning. Lower pretreatment cognitive reserve may make older patients more prone to the negative influence of chemotherapy on brain function, resulting in small cognitive changes in cognition being detectable more easily with neuropsychological tests [34]. Although Ahles et al. [32] corrected for cognitive reserve at baseline, they did not find a significant relation with older age and self-reported subjective cognitive function, solely chemotherapy treatment was found to be significantly related to self-reported subjective cognitive function. Mandelblatt et al. [29] recently showed that subjective self-reported cognition for survivors (mean age 68) exposed to chemotherapy decreases over time, whereas cognition of non-exposed patients did not change; however, the decline among the chemo exposed was non-significant. They found that older patients with genotypes associated with neurodegenerative disease, such as apolipoprotein E, exposed to chemotherapy showed a clinically meaningful decrease in self-reported cognitive function at 24 months [29]. These results support the idea that not only aging but also aging phenotypes are associated with lower self-reported cognitive function. It has been suggested that chemotherapy can lead to cancer-related cognitive declines through acceleration of aging processes [35].

Our study indicates that chemotherapy predominantly has a substantial detrimental effect on subjective self-reported cognition in younger women (particularly < 55 years). Janelsins et al. compared subjective self-reported cognitive function of patients exposed to chemotherapy (*n* = 581), mean age of 53 years, to age-matched non-cancer controls. Results showed that self-reported cognitive impairment is a substantial and pervasive problem for patients during and after chemotherapy treatment [28]. Younger age and black race were associated with problems with perceived cognitive abilities [28]. As cancer treatment, like chemotherapy, may accelerate the aging process via processes like DNA damage, it is plausible that the greatest effect of chemotherapy on cognitive function is detected in younger patients [36]. Furthermore, it might be that younger patients may detect impairment more readily as they are less likely to attribute problems to age-related changes. Also, such subjective cognitive problems are less likely to be obscured by preexisting age-related deficits [31]. Younger women may perceive subjective cognitive impairment due to treatment more often than older patients because they are more often employed and working, which requires more from their cognition and intellect.

Besides chemotherapy, other factors may also affect subjective cognitive functioning in patients. Also, in women ≤ 65 years treated without chemotherapy, cognitive functioning was lower compared to healthy age-matched controls. The impact of a cancer diagnosis itself, surgery, and toxicity due to other treatments may also have effect on the reporting of cognitive symptoms [37]. Furthermore, subjective cognitive changes are strongly associated with other patient-reported outcomes like fatigue, anxiety, and depression making it challenging to determine whether cognitive functioning is solely related to underlying brain dysfunction [11]. Longitudinal study in 187 breast cancer patients with and without chemotherapy exposure from Hermelink et al., reported that psychological consequences of a cancer diagnosis and treatment contribute more to cognitive dysfunction than the effects of medication [37]. The feeling of life disruption and sick leave might be more pronounced in younger patients contributing to greater cognitive impairment compared to older patients [37].

Sensitivity analysis was performed stratifying by menopausal status instead of age. Reason behind this is that evidence has emerged suggesting that hormonal changes, alone or in combination with chemotherapy and endocrine therapy, may cause cognitive impairment [29, 38–40]. An observed effect could thus be an age-related effect rather than an independent effect of systemic treatment per se. Results showed that the effect of chemotherapy was largest in pre/perimenopausal women treated with chemotherapy.

This study adds to the growing literature suggesting that patients experience subjective self-reported cognitive problems after cancer and/or cancer-related treatments like chemotherapy. The effect of chemotherapy on subjective cognitive function, especially in young patients at 6, 12, and 18 months after baseline, emphasizes the importance of adequate selection of early-stage breast cancer patients whom benefit most from chemotherapy treatment and to avoid overtreatment with chemotherapy and its attendant toxic effects. In recent years, research has been devoted to the development and validation of genomic tests that can provide not only prognostic information but perhaps more importantly can predict response to therapy [41]. Oncotype DX and MammaPrint are well validated and the most widely used multigene signatures for predicting outcomes in breast cancer [42, 43]. The use of genomic testing to guide chemotherapy treatment has been shown to lead to a reduction in the use of adjuvant chemotherapy in patients with early-stage breast cancer [42]. This highlights the importance of genomic testing to guide decisions on withholding chemotherapy in selected patients. Furthermore, results of this study, can help patients and doctors in shared decision making about chemotherapy treatment. As many young patients have to return to work, they should be adequately informed by physicians about the risks of cognitive decline during and after treatment with chemotherapy.

A limitation of our study is the fact that the UMBRELLA cohort was not specifically designed to evaluate the effect of chemotherapy, and patients were enrolled in our study at their first consultation with the radiation oncologist instead. In the majority of the patients, this was after breast surgery before the start of radiotherapy treatment. However, not all patients were enrolled and completed the baseline PRO questionnaires, at the same time in their treatment trajectory (i.e., after surgery, before the start of radiotherapy). This especially applies for women who had received neoadjuvant chemotherapy treatment and were enrolled before starting radiotherapy treatment which were mostly younger patients. Although we tried to correct for these differences in treatment trajectories, we might have missed early psychological complaints due to cancer diagnosis and neoadjuvant chemotherapy in some patients. Furthermore, the lack of pretreatment subjective cognitive function, which prevented us from adjusting for pretreatment cognitive function, may determine the amount of cognitive decline. The use of the EORTC Cognitive Functioning alone, to assess subjective self-reported cognitive function, may result in an underestimation of the extent of an individual’s cognitive symptoms as this EORTC domain encompasses and only captures two aspects of cognition (concentration and memory) [11]. Also, self-assessment of cognitive functioning may be prone to nocebo effects. In other words, information about the association between cognitive difficulties and chemotherapy or endocrine therapy given to chemotherapy patients and patients who received endocrine therapy (± chemotherapy) might increase the reporting of cognitive problems [44]. No formal neuropsychological tests were performed to test objective cognitive functioning. Objective cognitive functioning might have provided us with a different association between chemotherapy and the effect on cognition since self-reported cognitive symptoms are known to be strongly associated with other patient-reported outcomes like fatigue, anxiety, and depression [11]. Although we corrected for the use of endocrine therapy, we do not have information on compliance to adjuvant endocrine treatment. A substantial proportion of patients may have not been compliant to endocrine treatment due to side effects.

In conclusion, patients treated with chemotherapy report more impaired subjective cognitive functioning as compared to those treated without chemotherapy. This effect starts shortly after treatment and is seen up until 24 months of follow-up. The association between chemotherapy and impaired subjective cognitive functioning is most pronounced in younger women, while in older patients differences in subjective cognitive function between exposed and non-exposed to chemotherapy were less. These results highlight the careful consideration that is needed in the current clinical decision making whether chemotherapy should be administered to patients with early-stage breast cancer. Furthermore, with the emergence of increasing evidence for the efficacy of cognitive training strategies, there should be more management options available to cancer patients to address this important issue.

## Electronic supplementary material

Below is the link to the electronic supplementary material.

Supplementary material 1 (DOCX 24 KB)
