# Supplementary material for: The effect of chemotherapy on subjective cognitive function in younger early-stage breast cancer survivors treated with chemotherapy compared to older patients
Source: Breast Cancer Res Treat. 2019 Feb 12;175(2):429–41. doi: 10.1007/s10549-019-05149-4 (PMC6533221; doi:10.1007/s10549-019-05149-4)
Supplement: Supplementary file 1 — Supplementary material 1 (DOCX 24 KB) [file 10549_2019_5149_MOESM1_ESM.docx]

| **Supplement Table 1.** Demographics of the Dutch reference population. | |
| --- | --- |
|  | **No. of women (%)** |
| **Total no. of women** | 944 (100) |
| **Age categories (age in years)** |  |
| <55 years | 494 (52) |
| 55-65 years | 247 (26) |
| >65 years | 203 (22) |
| **Marital status** |  |
| Single | 170 (18) |
| Married | 644 (68) |
| Other | 130 (14) |
| **Educational level**† |  |
| Low | 80 (8) |
| Medium | 480 (51) |
| High | 384 (41) |
| **HADS score on the Anxiety subscale,** mean (SD) | 3.3 (3.1) |
| **HADS score on the Depression subscale,** mean (SD) | 3.5 (3.0) |
| NOTE. Categories may not sum to total N because of missing values  † Lower educational level: Secondary school +/- elementary school, Medium educational level: lower vocational education and community college, Higher educational level: University  HADS; Hospital Anxiety and Depression scale. | |

| **Supplement table 2.** Demographics and disease characteristics of non-responders and responders to at baseline and at least one follow-up questionnaire participating in the UMBRELLA breast cancer cohort. | | |
| --- | --- | --- |
|  | **Non-responders**  **No. of patients (%)** | **Responders**  **No. of patients (%)** |
| **Total no. of patients** | 264 (27) | 715 (73) |
| **Age in years at inclusion**, median (IQR) | 54 (15) | 55 (13) |
| **Age category** |  |  |
| <55 years | 139 (53) | 355 (50) |
| 55-65 years | 90 (34) | 240 (34) |
| >65 years | 35 (13) | 120 (17) |
| **Menopausal status at inclusion** |  |  |
| Premenopausal | 80 (30) | 216 (30) |
| Peri- or postmenopausal | 163 (62) | 452 (63) |
| Unknown | 21 (8) | 47 (7) |
| **Pathological tumor stage** |  |  |
| T0 | 19 (7) | 47 (7) |
| T1 | 169 (64) | 497 (70) |
| T2 | 61 (23) | 136 (19) |
| T3 | 5 (2) | 20 (3) |
| Unknown | 10 (4) | 15 (2) |
| **Course of chemotherapy treatment** |  |  |
| Neo-adjuvant chemotherapy † | 76 (29) | 135 (19) |
| Adjuvant chemotherapy | 100 (38) | 226 (32) |
| **Surgical treatment** |  |  |
| Breast conserving surgery | 214 (81) | 622 (87) |
| Mastectomy | 50 (19) | 93 (13) |
| **Most invasive axillary procedure** |  |  |
| Sentinel lymph node biopsy | 219 (83) | 627 (88) |
| Axillary lymph node dissection | 34 (13) | 65 (9) |
| Unknown | 11 (4) | 23 (3) |
| **Estrogen receptor status** |  |  |
| Negative | 55 (21) | 110 (15) |
| Positive‡ | 209 (79) | 603 (84) |
| Unknown | 0 (0) | 2 (0) |
| **HER2 receptor status** |  |  |
| Negative | 217 (82) | 608 (85) |
| Positive | 46 (17) | 97 (14) |
| Unknown | 1 (0) | 7 (1) |
| **Adjuvant chemotherapy treatment** |  |  |
| No | 164 (62) | 488 (68) |
| Yes | 100 (38) | 226 (32) |
| **Endocrine treatment** |  |  |
| No | 108 (41) | 328 (46) |
| Yes | 156 (59) | 387 (54) |
| **Type of radiotherapyⱡ** |  |  |
| Local radiotherapy | 171 (65) | 548 (77) |
| Locoregional radiotherapyⱡⱡ | 93 (35) | 166 (23) |
| Partial breast | 0 (0) | 1 (0) |
| NOTE. Categories may not sum to total N or 100% because of missing values and rounding.  † Neo-adjuvant chemotherapy was given in combination with immunotherapy if patients were HER2 receptor positive.  ‡ Estrogen receptor positive >10%  ⱡ Radiotherapy on the breast or chest wall with or without boost on the tumorbed  ⱡⱡ Includes radiotherapy on axillary and/or periclavicular lymph nodes and/or internal mammary nodes  Abbreviations: IQR, interquartile range; NA, not applicable; HER2, human epidermal growth factor receptor 2. | | |

| **Supplement Table 3.** Cognitive function in breast cancer patients by menopausal status at baseline assessed with the EORTC QLQ-C30 at baseline, three, six, twelve-, eighteen-, and twenty-four-months after enrollment in the UMBRELLA cohort. The difference in mean score (MD) between are adjusted for educational level, tumor stage, endocrine treatment and anxiety and depression and show the difference in cognitive function between chemotherapy and non-chemotherapy treatment. | | | | | | | | | | |
| --- | --- | --- | --- | --- | --- | --- | --- | --- | --- | --- |
|  |  |  | Pre/perimenopausal status | | |  |  | Postmenopausal status | | |
| No chemotherapy (reference) |  |  | n =87 | | |  |  | n = 240 | | |
| Chemotherapy |  |  | n =191 | | |  |  | n = 150 | | |
|  |  | MD^a^ | 95% CI | *P* value^b^ | ES^c^ |  | MD^a^ | 95% CI | *P* value^b^ | ES^c^ |
| Baseline |  | 8.4 | 4.3-12.4 | 0.000 | 0.4 |  | 2.4 | -3.5-8.3 | 0.427 | 0.1 |
| 3 Months |  | 8.1 | 3.8-12.5 | 0.000 | 0.4 |  | 5.6 | 1.7-9.4 | 0.005 | 0.2 |
| 6 Months |  | 13.5 | 8.8-18.2 | 0.000 | 0.6 |  | 5.1 | 1.0-9.2 | 0.014 | 0.2 |
| 12 Months |  | 11.4 | 6.6-16.2 | 0.000 | 0.5 |  | 6.6 | 2.3-10.8 | 0.002 | 0.3 |
| 18 Months |  | 11.6 | 6.1-17.1 | 0.000 | 0.4 |  | 5.5 | 1.0-10.1 | 0.017 | 0.2 |
| 24 Months |  | 11.0 | 4.4-17.6 | 0.001 | 0.4 |  | 5.4 | 0.2-10.6 | 0.040 | 0.2 |
| Patient reported outcomes on cognitive functioning according to EORTC QLQ-C30.  Between-group effects were assessed using mixed models including the measurements obtained at baseline and at 3, 6, 12, 18 and 24 months, adjusted for multiple testing, tumor stage, endocrine treatment, educational level, anxiety (HADS) and depression (HADS). Patients not exposed to chemotherapy serve as a reference category to calculate mean differences.  Abbreviations: N = number of patients; MD = mean difference; 95% CI = confidence interval; ES = effect size.  ^a^ Difference in mean score with reference group.  ^b^ The *P* value shown for the difference between no chemotherapy versus chemotherapy group.  ^c^ Standardized effect size calculated (mean difference divided by the pooled standard deviation) as a measure for minimal clinically important change. Small effect if ES 0.2-0.4, medium 0.5-0.7, large effect >0.8. | | | | | | | | | | |

| **Supplement Table 4.** Cognitive function in younger (< 55 years), middle-aged (55-65 years) and older (>65 years) patients with breast cancer assessed with the EORTC QLQ-C30 at baseline, three, six, twelve, eighteen and twenty-four months after first consultation with the radiation oncologist. The difference in mean score (MD) between in the younger, middle-aged and older patient group are adjusted for endocrine treatment and show the difference in cognitive function between chemotherapy and non-chemotherapy treatment excluding patients treated with neoadjuvant chemotherapy. | | | | | | | | | | | | | | | | |
| --- | --- | --- | --- | --- | --- | --- | --- | --- | --- | --- | --- | --- | --- | --- | --- | --- |
|  |  | Age <55 years | | | |  | Age 55-65 years | | | |  | Age >65 years | | | |  |
| No chemotherapy *(reference group)* |  | n = 128 | | | |  | n = 133 | | | |  | n = 93 | | | |  |
| Chemotherapy |  | n = 125 | | | |  | n = 82 | | | |  | n = 20 | | | |  |
|  |  | MD^a^ | 95% CI | *P* value^b^ | ES^c^ |  | MD^a^ | 95% CI | *P* value^b^ | ES^c^ |  | MD^a^ | 95% CI | *P* value^b^ | ES^c^ |  |
| Baseline |  | 12.9 | 3.7-22.0 | 0.006 | 0.4 |  | 2.4 | -15.3-20.4 | 0.790 | 0.1 |  | 3.7 | -16.6-20.1 | 0.812 | 0.0 |  |
| 3 Months |  | 4.8 | 0.6-8.9 | 0.024 | 0.2 |  | 4.8 | 0.8-9.4 | 0.004 | 0.3 |  | 8.7 | -4.3-19.7 | 0.092 | 0.3 |  |
| 6 Months |  | 14.0 | 9.6-18.5 | 0.000 | 0.6 |  | 3.6 | -1.4-8.6 | 0.162 | 0.2 |  | -3.7 | -16.8-7.5 | 0.629 | 0.2 |  |
| 12 Months |  | 12.2 | 7.6-16.8 | 0.000 | 0.5 |  | 8.1 | 2.0-13.3 | 0.002 | 0.4 |  | -1.2 | -14.2-10.5 | 0.877 | 0.1 |  |
| 18 Months |  | 13.7 | 7.9-18.2 | 0.000 | 0.5 |  | 6.8 | 1. -12.4 | 0.019 | 0.3 |  | -11.1 | -23.3-3.6 | 0.085 | 0.4 |  |
| 24 Months |  | 8.8 | 2.8-14.8 | 0.004 | 0.4 |  | 9.1 | 2.8-15.3 | 0.005 | 0.4 |  | -10.2 | -25.1-6.1 | 0.245 | 0.3 |  |
| Patient reported outcomes on cognitive functioning according to EORTC QLQ-C30.  Between-group effects were assessed using mixed models including the measurements obtained at baseline and at 3, 6, 12, 18 and 24 months, adjusted for multiple testing, tumor stage, endocrine treatment, educational level, anxiety (HADS) and depression (HADS). Patients not exposed to chemotherapy serve as a reference category to calculate mean differences.  Abbreviations: N = number of patients; MD = mean difference; 95% CI = confidence interval; ES = effect size.  ^a^ Difference in mean score with reference group.  ^b^ The *P* value shown for the difference between no chemotherapy versus chemotherapy group.  ^c^ Standardized effect size calculated (mean difference divided by the pooled standard deviation) as a measure for minimal clinically important change. Small effect if ES 0.2-0.4, medium 0.5-0.7, large effect >0.8.  ^d^ The *P* value shown for the interaction Age-by-time by treatment (no chemotherapy versus chemotherapy). | | | | | | | | | | | | | | | | |
